# Фенотипический состав Т-лимфоцитов периферической крови у пациентов с болезнью Грейвса при длительной консервативной терапии тиамазолом

**DOI:** 10.14341/probl12812

**Published:** 2021-12-30

**Authors:** М. А. Дудина, С. А. Догадин, А. А. Савченко, В. Д. Беленюк

**Affiliations:** Красноярский государственный медицинский университет имени профессора В.Ф. Войно-Ясенецкого; Краевая клиническая больница; Красноярский государственный медицинский университет имени профессора В.Ф. Войно-Ясенецкого; Краевая клиническая больница; Красноярский государственный медицинский университет имени профессора В.Ф. Войно-Ясенецкого; Красноярский научный центр Сибирского отделения Российской академии наук», обособленное подразделение «НИИ медицинских проблем Севера»; Красноярский научный центр Сибирского отделения Российской академии наук», обособленное подразделение «НИИ медицинских проблем Севера»

**Keywords:** Болезнь Грейвса, Т-регуляторные клетки, активированные Т-хелперы, иммуномодулирующие эффекты тиреостатических препаратов, таргетная терапия

## Abstract

**ОБОСНОВАНИЕ:**

ОБОСНОВАНИЕ. Эффективный контроль аутоиммунного воспаления при болезни Грейвса предопределяет необходимость изучения дисфункции хелперных и цитотоксических Т-лимфоцитов, а также степени активации регуляторных Т-клеток при тиреостатической терапии болезни Грейвса, что позволит уточнить иммуномодулирующие эффекты длительной консервативной терапии тиамазолом и определить мишени для разработки современной таргетной терапии.

**ЦЕЛЬ:**

ЦЕЛЬ. Изучить фенотипический состав Т-лимфоцитов периферической крови у пациентов с болезнью Грейвса для оценки направленности иммунного ответа в зависимости от длительности медикаментозного эутиреоза.

**МАТЕРИАЛЫ И МЕТОДЫ:**

МАТЕРИАЛЫ И МЕТОДЫ. Проведено одноцентровое одномоментное когортное сплошное открытое контролируемое исследование с оценкой фенотипического состава Т-клеток в периферической крови у женщин с болезнью Грейвса. Методом проточной цитометрии с использованием прямой иммунофлуоресценции с применением моноклональных антител были исследованы особенности фенотипа T-лимфоцитов в зависимости от продолжительности медикаментозного эутиреоза при консервативной терапии тиамазолом.

**РЕЗУЛЬТАТЫ:**

РЕЗУЛЬТАТЫ. В исследование включены 135 женщин с верифицированным диагнозом болезни Грейвса, средний возраст 43,09±12,81 года, из них 120 (88,91%) — с рецидивом заболевания и 15 (11,09%) — с впервые выявленным гипертиреозом. Установлено повышение процентного содержания активированных Т-хелперов (CD3+CD4+CD25+) у  больных болезнью Грейвса с продолжительностью медикаментозного эутиреоза от 5 до 8 мес и от 9 до 12 мес соответственно, Me=0,94 (0,48–1,45; p=0,020) и Me=0,95 (0,41–1,80; p=0,025), у лиц контрольной группы — Ме=0,12 (0,03-0,68). Установлено повышение количества регуляторных Т-лимфоцитов (CD4+CD25+CD127Low) как в группе больных с продолжительностью медикаментозного эутиреоза от 5 до 8 мес (Me=3,01 (1,88–4,47); p=0,024), так и у пациентов с длительностью медикаментозного эутиреоза от 9 до 12 мес (Me=5,52 (2,77–11,61); p<0,001) в сравнении с показателями группы контроля (Ме=1,81 (0,91-2,82)). Уровень регуляторных Т-клеток в периферической крови пациентов с болезнью Грейвса с продолжительностью медикаментозного эутиреоза более 12 мес снижается, но сохраняется повышенным относительно контроля.

**ЗАКЛЮЧЕНИЕ:**

ЗАКЛЮЧЕНИЕ. У пациентов с болезнью Грейвса с продолжительностью медикаментозного эутиреоза от 5 до 8 мес и от 9 до 12 мес повышается популяция регуляторных Т-лимфоцитов с фенотипом CD4+CD25+CD127Low. Количество активированных Т-хелперов с фенотипом CD3+CD4+CD25+ сохраняется повышенным независимо от продолжительности медикаментозного эутиреоза. У пациентов с болезнью Грейвса с продолжительностью медикаментозного эутиреоза более 12 мес сохраняется компенсаторное повышение регуляторных Т-лимфоцитов, а общее количество Т-хелперов восстанавливается до уровня контроля.

## Abstract (en)

**BACKGROUND:**

BACKGROUND: Effective control of autoimmune inflammation in Graves’ disease determines necessity to study the T helper (Th) and cytotoxic T-lymphocytes dysfunction, as well as the level of regulatory T-cells (Treg) activation in patients with Graves’ disease on thyrostatic medication, which will clarify the immunomodulatory effects of long-term thiamazole treatment serve as targets for more specific therapies.

**AIM:**

AIM: To study the phenotypic composition of T-lymphocytes in the peripheral blood of patients with Graves’ disease to assess the direction of immune response depending on thimazole-induced euthyroidism duration.

**MATERIALS AND METHODS:**

MATERIALS AND METHODS: A single-center, cohort, continuous, open-label, controlled trial was conducted to assess the phenotypic composition of T-lymphocytes in peripheral blood in women with Graves’ disease on long-term thiamazole treatment. The phenotypic composition of T-lymphocytes was determined by flow cytometry using direct immunofluorescence with conjugated FITC monoclonal antibodies depending on the duration of thimazole-induced euthyroidism of long-term thiamazole treatment.

**RESULTS:**

RESULTS: The study included 135 women with Graves’ disease, mean age 43.09±12.81 years, 120 (88.91%) with a relapse of the disease and 15 (11.09%) with newly diagnosed hyperthyroidism. An increase of activated CD3+CD4+CD25+ was found in patients with Graves’ disease with a duration of thimazole-induced euthyroidism 5–8 months and 9–12 months, respectively, Me=0.94 (0.48–1.45), p=0.020) and Me=0.95 (0.41–1.80), p=0.025), in control group — Me=0.12 (0.03–0.68). Compared to the control an increase of CD4+CD25+CD127Low (Treg) was found in patients with a duration of thimazole-induced euthyroidism 5–8 and 9–12 months. The content of Treg in peripheral blood in Graves’ disease patients with a duration of thimazole-induced euthyroidism more than 12 months decreases, but remains elevated relative to the control.

**CONCLUSION:**

CONCLUSION: In patients with Graves’ disease with a duration of thimazole-induced euthyroidism 5–8 months and 9–12 months the level of Treg has been increased. The increase of activated Th (CD3+CD4+CD25+) persists independently of thimazole-induced euthyroidism. In patients with Graves’ disease with a duration of thimazole-induced euthyroidism for more than 12 months, there is a compensatory increase in regulatory T-lymphocyte, and the total number of T-helpers is restored to the control.

## ОБОСНОВАНИЕ

Болезнь Грейвса характеризуется развитием тиреотоксикоза, вызванного циркуляцией антител к рецептору тиреотропного гормона (рТТГ), которые усиливают индуцируемую интерфероном-гамма (ИФН-γ) экспрессию молекул 2-го класса гистосовместимости (HLA-DR) и презентацию тиреоидных антигенов, способствуя дальнейшей активации Т-хелперов (Th), что клинически может проявляться как стимуляцией, так и ингибированием функции щитовидной железы [1]. В последнее время изменился характер течения гипертиреоза аутоиммунного генеза. Отмечаются выраженное ускорение манифестации заболевания у предрасположенных лиц, резкое снижение количества пациентов с ремиссией тиреотоксикоза и ее продолжительностью на фоне консервативной терапии тиреостатиками [2].

Механизм иммуносупрессивного действия тиреостатических препаратов остается предметом споров, несмотря на многочисленные утверждения о том, что тионамиды напрямую ингибируют йодирование тиреоглобулина, что может повлиять на презентацию антигенов тиреоцитами, а также препятствуют экспрессии в тиреоцитах таких молекул, как интерлейкин-1 (IL-1), интерлейкин-6 (IL-6), простагландин Е2 и белок теплового шока [3]. Однако в ряде клинических исследований у пациентов с болезнью Грейвса отмечаются снижение активации СD4+ Т-лимфоцитов и повышение количества CD8+ цитотоксических Т-лимфоцитов, а также понижение уровня растворимых рецепторов IL-2, что определяется достижением стойкого медикаментозного эутиреоза при длительной тионамидной терапии метимазолом [4][5].

В настоящее время имеются предположения о том, что Т-хелперный иммунный ответ 1-го типа (Th1) преобладает в иммунопатогенезе болезни Грейвса во время активной фазы гипертиреоза, в которой хемокины Th1 и их рецептор CXCR3 играют ключевую роль [6]. Эти нарушения считаются результатом первичного нарушения иммунорегуляции, при которой дисфункция щитовидной железы обуславливается агрессией сохранивших активность Th1- и Th2-лимфоцитов против специфических антигенов клеток-мишеней. Остаются до сих пор неизвестными влияния тиреостатической терапии на дефект недостаточной активации регуляторных Т-клеток и формирование супрессорных механизмов в иммунной системе при болезни Грейвса, что не только определяет степень дисфункции тиреоидспецифических хелперных Т-лимфоцитов, но и может непосредственно индуцировать переход от доминирования Th2- к доминированию Th1-иммунного ответа в клиническом течении заболевания.

В связи с этим эффективный контроль аутоиммунного воспаления при болезни Грейвса предопределяет необходимость уточнения иммунологических критериев прогрессирования заболевания на фоне тиреостатической терапии для разработки современной стратегии таргетной терапии.

## ЦЕЛЬ ИССЛЕДОВАНИЯ

Изучить фенотипический состав Т-лимфоцитов периферической крови у пациентов с болезнью Грейвса для оценки направленности иммунного ответа в зависимости от длительности медикаментозного эутиреоза.

## МАТЕРИАЛЫ И МЕТОДЫ

Место и время проведения исследования

Место проведения. Исследование проводилось на базе эндокринологического центра КГБУЗ «Краевая клиническая больница» (г. Красноярск).

Время исследования. Наблюдение пациентов осуществлялось с 9 апреля 2016 г. по 10 апреля 2021 г.

Изучаемые популяции (одна или несколько)

Изучались две популяции: пациенты с болезнью Грейвса и здоровые индивиды (контроль).

Популяция «пациенты с болезнью Грейвса»

Критерии включения: женский пол, возраст от 18 до 65 лет, лабораторно подтвержденная болезнь Грейвса, стойкий медикаментозный эутиреоз, индуцированный непрерывным приемом тиамазола.

Популяция «контроль»

Критерии включения: женский пол, возраст от 18 до 65 лет, отсутствие отягощенного анамнеза по заболеваниям щитовидной железы у обследуемой пациентки и кровных родственников, отсутствие структурных изменений по данным ультразвукового исследования (УЗИ) щитовидной железы.

Критерии исключения: узловой/многоузловой токсический зоб, беременность, лактация, эндокринная офтальмопатия, рецидив гипертиреоза после лечения болезни Грейвса радиоактивным йодом, наличие инфекционных и аллергических заболеваний, новообразования, системные заболевания соединительной ткани, другие заболевания органов эндокринной системы, острые респираторные и вирусные инфекции, а также введение профилактических прививок в течение 2 мес, предшествующих иммунологическому и гормональному анализу.

Способ формирования выборки из изучаемой популяции (или нескольких выборок из нескольких изучаемых популяций)

В основу формирования подгрупп пациентов с болезнью Грейвса была положена концепция иммуномодулирующего эффекта тиреостатической терапии при условии стойкого медикаментозного эутиреоза, индуцированного непрерывным приемом тиамазола, использовавшегося по стандартному протоколу консервативного лечения болезни Грейвса [7]. Выбор сроков для клинико-иммунологического обследования пациентов с болезнью Грейвса базировался на стабильности достигнутого медикаментозного эутиреоза. Стойкая нормализация гормонов щитовидной железы включала, в том числе, стабилизацию уровня ТТГ в пределах нормального референсного диапазона. Предварительный анализ показал, что сроки поддержания состояния стойкого медикаментозного эутиреоза на фоне непрерывного приема тиамазола у обследуемых пациентов с болезнью Грейвса варьируют от 5 до 12 мес и более. В связи с этим пациентки со стойким медикаментозным эутиреозом при болезни Грейвса были разделены на репрезентативные подгруппы внутри указанного периода наблюдения, а также с учетом рекомендуемых сроков консервативного лечения заболевания согласно национальным клиническим рекомендациям. Изучаемая популяция пациентов с болезнью Грейвса была разделена на три группы: первая — продолжительность медикаментозного эутиреоза от 5 до 8 мес, вторая — продолжительность медикаментозного эутиреоза от 9 до 12 мес, третья — продолжительность медикаментозного эутиреоза более 12 мес.

Дизайн исследования

Проведено одноцентровое одномоментное когортное сплошное открытое контролируемое исследование с ретроспективным анализом данных с участием пациентов с лабораторно подтвержденной болезнью Грейвса.

Методы

Клинические методы обследования на момент рандомизации включали объективный осмотр, пальпацию щитовидной железы, клинико-лабораторную оценку тиреоидного статуса и титра антител к рТТГ в сыворотке крови. Изучение клинико-анамнестических данных обследуемых пациентов с впервые выявленным или рецидивирующим течением заболевания проводилось с использованием медицинской документации (амбулаторные карты, форма № 025/y-04; истории болезней стационарного больного, форма № 003/у).

При этом оценивали хронологию развития заболевания с уточнением времени от момента манифестации гипертиреоза, верификации диагноза болезни Грейвса и инициации тиреостатической терапии, дозу тиамазола, гормональные показатели, титр антител к рТТГ исходно и на фоне медикаментозного лечения, а также продолжительность тиреостатической терапии. Методом хемилюминесцентного иммуноанализа на микрочастицах на автоматическом анализаторе Architecti1000sr (Abbott Diagnostics, США) определяли уровень ТТГ и свободного тироксина (св.Т4) в сыворотке крови, указанные референсные диапазоны соответственно 0,4–4,0 мЕд/л и 9,01–19,05 пмоль/л. Определение свободного трийодтиронина (св.Т3) в сыворотке крови осуществляли методом энзим-связанного иммуноферментного анализа (ИФА) с использованием тест-систем «ДС-ИФА-ТИРОИД-Т3свободный» (ООО «НПО «Диагностические системы», Россия), референсный интервал 2,14–6,42 пмоль/л. Уровень антител к рТТГ оценивался методом ИФА при помощи стандартного набора Medizym T.R.A. (MedipanDiagnostica, Германия), рекомендованная точка разделения (cut-off) — 1,5 мЕд/л («серая» зона 1–1,5 мЕд/л). Уровень антител к тиреоидной пероксидазе (ТПО) оценивался методом ИФА при помощи соответствующего набора «АТ-ТПОХема-Медика» (Россия), референсный интервал <35 мЕд/л. Определение размеров, объема и структуры щитовидной железы проводилось на основании УЗИ на аппарате Aloka 3500 (Hitachi, Япония) с использованием линейного датчика с частотой 7,5 МГц.

Исследование фенотипа Т-лимфоцитов проводили методом проточной цитометрии с использованием прямой иммунофлуоресценции с применением моноклональных антител (Beckman Coulter, США), меченных FITC (fluoresceinisothiocyanate), PE (phycoerythrin), ECD (phycoerythrin-TexasRed-X), PC5 (phycoerythrin-cyanin 5), PC7 (phycoerythrin-cyanin 7) и APC (allophycocyanin) в следующей панели: CD45-FITC/CD127-PE/CD3-ECD/CD25-PC5/CD4-PC7/CD8-APC. Анализ окрашенных клеток проводили на проточном цитофлуориметре Navios (Beckman Coulter, USA) Центра коллективного пользования КНЦ СО РАН. Обработку полученных цитофлуориметрических результатов осуществляли с помощью программ Navios Software v. 1.2 и Kaluza v. 2.1.1 (Beckman Coulter, USA). В каждой пробе анализировали не менее 50 000 лимфоцитов.

Статистический анализ

Описание полученных данных производили с помощью подсчета медианы (Ме) и квартильного размаха в виде 1 и 3 квартилей (Q1–Q3), а также в виде средних арифметических значений и стандартных отклонений от среднего (М±SD) в случае нормального распределения показателей. Для определения характера распределения полученных данных использовали критерий Шапиро–Уилка. Достоверность различий между исследуемыми показателями оценивали по непараметрическому критерию Манна–Уитни (Mann–Whitney U-test). Для исследования силы взаимосвязей показателей вычислялся коэффициент ранговой корреляции по Спирмену (Spearman rank R). Статистический анализ осуществляли в пакете прикладных программ Statistica 8.0 (StatSoft Inc., 2007).

Этическая экспертиза

Протокол исследования одобрен Локальным этическим комитетом КГБУЗ «Краевая клиническая больница» от 07 апреля 2016 г. (выписка из протокола № 124) и ФБОУ ВО «КрасГМУ» от 9 ноября 2016 г. (выписка из протокола № 72/2016).

## РЕЗУЛЬТАТЫ

В исследование были включены 135 женщин с верифицированным диагнозом болезни Грейвса, средний возраст 43,14±12,84 года, из них 120 (88,9%) — с рецидивом заболевания и 15 (11,1%) — с впервые выявленным гипертиреозом. В качестве контроля обследованы 85 неродственных, практически здоровых женщин.

Все обследованные пациенты с болезнью Грейвса наблюдались в исследовательском центре с дебюта заболевания. При ретроспективном анализе показателей тиреоидного статуса в дебюте заболевания было установлено, что у обследованных пациентов с болезнью Грейвса I и II групп был диагностирован субклинический или манифестный гипертиреоз соответственно, ТТГ=0,07 мЕд/л (0,03–0,39), св.Т3=5,92 пмоль/л (4,61–6,41), св.Т4=17,33 пмоль/л (12,19–18,77) и ТТГ=0,05 мЕд/л (0,01–0,37), св.Т3=6,51 пмоль/л (4,31–7,43), св.Т4=21,52 пмоль/л (19,11–36,77). У большинства пациентов III группы показатели тиреоидного статуса в дебюте заболевания соответствовали субклиническому гипертиреозу: ТТГ=0,03 мЕд/л (0,09–0,36), св.Т3=5,12 пмоль/л (3,92–6,31), св.Т4=16,41 пмоль/л (12,88–18,78). При ретроспективном сравнительном анализе исходных показателей тиреоидного статуса и уровня антител к рТТГ подгрупп пациентов с болезнью Грейвса, в дальнейшем обследованных в зависимости от продолжительности медикаментозного эутиреоза, статистически значимых различий не выявлено.

Независимо от продолжительности медикаментозного эутиреоза в периферической крови обследуемых пациентов с болезнью Грейвса в дебюте заболевания был повышен относительный уровень T-хелперов (CD3+CD4+), в том числе активированных (CD3+CD4+CD25+) соответственно, Ме=66,42% (44,23–74,61), p=0,011 и Ме=5,51% (4,12–6,73), p=0,013. Процентное количество регуляторных CD3+CD4+CD127LowCD25High-клеток в крови у всех обследуемых пациентов на момент манифестации болезни Грейвса было снижено — Ме=1,21% (0,92–1,81), p=0,021.

Клинико-гормональная характеристика обследуемых пациентов с болезнью Грейвса на момент включения в настоящее исследование представлена в таблице 1.

На момент клинико-иммунологического обследования у всех пациентов был достигнут индуцированный тиамазолом медикаментозный эутиреоз, что подтверждалось при объективном осмотре и соответствующими показателями тиреоидного статуса. Все обследуемые пациенты с болезнью Грейвса имели положительный титр антител к рТТГ, причем величина вышеуказанного маркера не различалась в зависимости от продолжительности медикаментозного эутиреоза. При этом максимальная продолжительность приема тиамазола в III группе больных составила 36 мес.

При исследовании фенотипа Т-лимфоцитов было обнаружено, что у пациентов с болезнью Грейвса абсолютное и относительное содержание Т-клеток (CD3+) в периферической крови не изменялось в зависимости от продолжительности медикаментозного эутиреоза и соответствовало контрольным значениям (табл. 2).

**Table:** Таблица 1. Клинико-гормональная характеристика пациентов с болезнью Грейвса по группам, Ме (Q1–Q3) Примечание. p1 — статистически значимые различия с контрольными величинами; p2 — статистически значимые различия с показателями пациентов I-й группы; p3 — статистически значимые различия с показателями пациентов II-й группы; св.Т4 — свободный тироксин; св.Т3 — свободный трийодтиронин; рТТГ — рецептор тиреотропного гормона; ТПО — тиреоидная пероксидаза.

Показатели	Контроль	Пациенты с болезнью Грейвса
n=85	Группа IМедикамент. эутиреоз5–8 месn=41	Группа IIМедикамент. эутиреоз9–12 месn=69	Группа IIIМедикамент. эутиреоз>12 месn=25
1	2	3	4
Возраст, лет (M±SD)	41,02±12,11	43,91±13,76	42,94±12,24	42,43±13,09
Продолжительность приема тиамазола, мес	–	7,0(6,0–8,0)	11,0(10,0–12,0)	16,0(14,0–24,0)
Доза тиамазола, мг	–	10,0(10–20)	10,0(10–20)	10,0(10–15)
Тиреотропный гормон, мЕд/л	1,13(0,86–1,51)	0,78(0,41–2,11)p1<0,001	1,09(0,55–3,43)p1<0,001	1,91(0,57–5,31)p1=0,004
Св.Т3, пмоль/л	4,07(2,61–5,53)	5,31(3,83–6,31)p1<0,001	4,73(3,82–5,93)p1<0,001	2,24(2,93–5,12)p1=0,027
Св.Т4, пмоль/л	14,10(12,28–15,80)	13,07(10,49–16,91)p1<0,001	12,83(10,19–16,97)p1<0,001	15,57(11,83–17,43)p1=0,022p3=0,030
Антитела к рТТГ, мЕд/л	0,24(0,18–0,43)	15,03(9,45–21,04)p1<0,001	15,27(9,45–21,45)p1<0,001	15,62(9,32–24,52)p1<0,001
Антитела к ТПО, мЕд/л	1,00(0,00–3,00)	223,21(13,01–563,11)p1<0,001	227,21(29,13–695,51)p1<0,001	632,00(78,00–1186,00)p1=0,003
Объем щитовидной железы, мл	9,91(9,41−12,63)	29,19(20,41–45,32)p1<0,001	32,21(20,51–49,88)p1<0,001	27,36(16,65–50,61)p1<0,001

**Table:** Таблица 2. Фенотипический состав Т-лимфоцитов в периферической крови пациентов с болезнью Грейвса (Ме, (Q1–Q3) Примечание. p1 — статистически значимые различия с контрольными величинами; p2 — статистически значимые различия с показателями пациентов I группы; p3 — статистически значимые различия с показателями пациентов II-й группы.

Показатели	Контроль	Пациенты с болезнью Грейвса
n=85	Группа IМедикамент. эутиреоз5–8 месn=41	Группа IIМедикамент. эутиреоз9–12 месn=69	Группа IIIМедикамент. эутиреоз>12 месn=25
1	2	3	4
CD3+, 109/л	1,51(1,19–1,65)	2,00(1,14–2,52)	1,59(1,17–2,03)	1,81(0,63–2,29)
CD3+, %	72,0(68,31–76,12)	77,6(69,21–84,62)	74,41(66,81–78,41)	70,95(58,91–75,49)
CD3+CD25+, %	0,21(0,03–1,70)	1,18(0,33–3,07)p1=0,045	1,41(0,66–2,52)p1=0,040p2=0,027	2,67(1,74–4,02)p1<0,001p2=0,038p3=0,043
CD3+CD4+, %	41,4(37,8–46,2)	52,7(38,4–55,4)p1=0,031	49,7(39,9–54,4)	40,1(34,4–48,3)p2=0,042
CD3+CD8+, %	30,1(26,2–35,1)	25,2(21,5–26,2)p1=0,014	22,3(20,1–28,7)p1<0,001	29,3(18,3–36,3)
CD3+CD4+CD25+, %	0,12(0,03–0,68)	0,94(0,48–1,45)p1=0,020	0,95(0,41–1,80)p1=0,025	1,65(0,78–2,97)p1<0,001p2=0,036p3=0,038
CD3+CD8+CD25+, %	0,06(0,03–0,17)	0,31(0,03–1,05)	0,33(0,12–0,94)p1=0,045	0,99(0,52–1,88)p1<0,001p2=0,043p3=0,032
CD3+CD4+CD127LowCD25High, %	1,81(0,91–2,82)	3,01(1,88–4,47)p1=0,024	5,52(2,77–11,61)p1<0,001	3,21(1,04–4,30)p1=0,048p3=0,039

При сравнительном анализе фенотипического состава Т-лимфоцитов периферической крови в зависимости от продолжительности медикаментозного эутиреоза наиболее выраженные изменения обнаружены в увеличении содержания Т-клеток с фенотипом CD3+CD25+, несущих маркер ранней активации. С увеличением интервала времени нахождения пациентов с болезнью Грейвса в состоянии медикаментозного эутиреоза количество активированных Т-лимфоцитов (CD3+CD25+) последовательно повышалось. Относительное количество Т-хелперов (CD3+CD4+) у пациенток с болезнью Грейвса и медикаментозным эутиреозом в течение 5–8 мес было увеличено в сравнении с контрольным диапазоном. При увеличении продолжительности медикаментозного эутиреоза уровень CD3+CD4+-клеток стал соответствовать контрольным значениям. А при длительности эутиреоза более 12 мес содержание Т-хелперов было значительно ниже, чем в I группе. Уровень цитотоксических Т-лимфоцитов (CD3+CD8+) был значительно ниже контрольных значений как в группе пациентов с продолжительностью медикаментозного эутиреоза 5–8 мес, так и 9–12 мес, тогда как в III группе больных (эутиреоз более 12 мес) содержание цитотоксических Т-клеток соответствовало контрольному диапазону. Относительное количество активированных Т-хелперов (CD3+CD4+CD25+) было повышено у пациентов с продолжительностью медикаментозного эутиреоза в течение 5–8 и 9–12 мес и еще более возрастало в группе больных с продолжительностью медикаментозного эутиреоза более 12 мес.

У пациентов I группы процентное содержание в крови Т-клеток с фенотипом CD3+CD8+CD25+ соответствовало контрольным значениям, а в III группе их уровень был значительно выше контрольного. В то же время в III группе (эутиреоз более 12 мес) количество активированных Т-хелперов (CD3+CD4+CD25+) было значительно выше как относительно контрольных значений, так и по сравнению с данными, выявляемыми у пациентов I и II групп (продолжительность эутиреоза 5–8 и 9–12 мес).

Относительное количество Т-регуляторных клеток (CD3+CD4+CD127LowCD25High) в периферической крови у пациентов с болезнью Грейвса в сравнении с контрольным диапазоном было повышено независимо от продолжительности медикаментозного эутиреоза. Однако у больных с продолжительностью эутиреоза более 12 мес наблюдалось снижение содержания Т-регуляторных клеток относительно уровня, выявленного при медикаментозном эутиреозе продолжительностью 9–12 мес.

## ОБСУЖДЕНИЕ

Эффективность тиреостатической терапии в индукции и поддержании ремиссии болезни Грейвса определяется уровнем экспрессии рТТГ, за счет которых реализуется механизм дальнейшей презентации тиреоидных антигенов и увеличения количества активированных Т-хелперов [8]. Длительная тиреостатическая терапия приводит к снижению титра циркулирующих антител к рТТГ, выраженности лимфоцитарной инфильтрации щитовидной железы, уменьшению антигенной презентации на поверхности фолликулярных клеток и развитию медикаментозного эутиреоза [9]. Однако наблюдение за пациентками с болезнью Грейвса, длительно принимающими тиамазол, показало, что уровень антител к рТТГ остается положительным на любой стадии процесса, независимо от продолжительности тиреостатической терапии, наличия гипертиреоза или медикаментозного эутиреоза, а также не связан с изменениями фенотипического состава Т-лимфоцитов.

Причем необходимо отметить, что у пациентов с болезнью Грейвса в зависимости от продолжительности медикаментозного эутиреоза меняется именно фенотипический состав Т-лимфоцитов, в то время как процентное и абсолютное количество общих Т-клеток в крови остается постоянным и не отличается от контрольных показателей. В то же время у обследованных больных с разной продолжительностью медикаментозного эутиреоза наблюдается определенная динамика изменения относительного количества Т-хелперов (CD3+CD4+) и цитотоксических Т-клеток (CD3+CD8+). Так, если у пациентов с продолжительностью медикаментозного эутиреоза в течение 5–8 мес в крови выявляются повышенный (относительно контрольных значений) уровень Т-хелперов и сниженное количество цитотоксических Т-лимфоцитов, то у больных с продолжительностью медикаментозного эутиреоза 9–12 мес содержание Т-хелперов нормализуется, тогда как количество цитотоксических Т-клеток остается пониженным. При стойком медикаментозном эутиреозе более 12 мес у пациентов с болезнью Грейвса в крови наблюдаются еще более пониженный уровень (относительно исходного, но в диапазоне контрольных значений) Т-хелперов и нормализация количества цитотоксических Т-клеток. Необходимо отметить, что именно в составе субпопуляции Т-хелперов находится фракция Т-регуляторных клеток, которая реализует функции супрессии иммунного ответа [10].

Отмечено, что при болезни Грейвса снижена функциональная активность или количество Т-регуляторных клеток [1][6]. В настоящем исследовании показано, что уже при поддержании стойкого медикаментозного эутиреоза в течение 5–8 мес наблюдается иммуномодулирующий эффект консервативной терапии тиамазолом, заключающийся в выраженном увеличении количества Т-регуляторных клеток. При большей длительности медикаментозного эутиреоза (9–12 мес) содержание данной фракции Т-лимфоцитов достигает максимума, тогда как при дальнейшем увеличении длительности медикаментозного эутиреоза (более 12 мес) их количество в крови снова снижается, но сохраняется компенсаторное повышение относительно контроля. Повышение доли регуляторных Т-лимфоцитов (CD3+CD4+CD127LowCD25High) в периферической крови у больных с продолжительностью медикаментозного эутиреоза более 12 мес свидетельствует о том, что длительное лечение тиамазолом способствует однонаправленным изменениям: развитию медикаментозного эутиреоза и частичному восстановлению супрессорного потенциала регуляторных Т-лимфоцитов. Можно предположить, что высокий уровень Т-регуляторных клеток у пациентов с болезнью Грейвса, независимо от стойкости медикаментозного эутиреоза, является компенсаторной иммунной реакцией, направленной на преодоление дефекта резистентности к супрессорному действию регуляторных Т-лимфоцитов, необходимых на любой стадии процесса постоянной антигензависимой активации Т-хелперов при болезни Грейвса.

Кроме оценки субпопуляционного состава Т-лимфоцитов у пациентов с болезнью Грейвса в зависимости от длительности медикаментозного эутиреоза, также было исследовано содержание активированных Т-хелперов и цитотоксических Т-лимфоцитов. В качестве активационного маркера был исследован CD25. Молекула CD25 представляет собой гликопротеин, являющийся низкоаффинным рецептором к интерлейкину-2 и экспрессирующийся на Т-регуляторных клетках, активированных Т-хелперах и цитотоксических Т-лимфоцитах [11]. Количество активированных Т-хелперов (CD3+CD4+CD25+) повышено уже в I группе больных (эутиреоз в течение 5–8 мес). Максимальный уровень Т-клеток с данным фенотипом выявляется во II группе (эутиреоз длительностью более 12 мес). Содержание активированных цитотоксических Т-клеток (CD3+CD8+CD25+) повышается у больных II группы (эутиреоз 9–12 мес) и также достигает максимума в III группе пациентов (эутиреоидное состояние в течение более 12 мес) консервативного лечения тиамазолом.

Таким образом, у пациентов с болезнью Грейвса в зависимости от продолжительности медикаментозного эутиреоза меняется фенотипический состав Т-лимфоцитов, но при сохранении абсолютного и процентного содержания общих Т-клеток в периферической крови. Причем изменения фенотипа Т-лимфоцитов при медикаментозном эутиреозе разной продолжительности нельзя однозначно характеризовать как иммуносупрессивные. У больных с наибольшей продолжительностью медикаментозного эутиреоза (более 12 мес) нормализуется количество Т-хелперов и цитотоксических Т-лимфоцитов. Но в то же время у всех обследуемых пациентов с болезнью Грейвса сохраняются характерные изменения начала иммунной реакции с ослаблением супрессии активированных Т-хелперов, не зависящие от дозы и продолжительности медикаментозного эутиреоза. Вероятно, при болезни Грейвса супрессия T-хелперов ослабляется в такой степени, что они в присутствии антигена выходят из-под контроля и активируются.

Следует подчеркнуть, что активированные Т-хелперы прямо влияют на тиреоциты, продуцируя цитокины, стимулируют цитотоксические клетки, что приводит к повреждению тиреоидной ткани, а также усиливают продукцию плазматическими клетками тиреоидных аутоантител, которые вносят дополнительный вклад в поддержание аутоиммунного воспаления [12]. Тем не менее у пациентов с болезнью Грейвса и продолжительностью медикаментозного эутиреоза от 9 до 12 мес и более общее число T-хелперов (CD3+CD4+) в периферической крови соответствует контрольным значениям, что свидетельствует о частичном подавлении чрезмерной активации хелперных Т-клеток вместе с увеличением продолжительности времени нахождения пациентов с болезнью Грейвса в состоянии медикаментозного эутиреоза при консервативном лечении тиамазолом.

В ранее проведенных нами исследованиях было показано, что у пациентов с болезнью Грейвса, непрерывно получающих тиамазол от 12 до 18 мес, содержание T-хелперов с фенотипами CD3+CD4+- и CD3+CD4+CD25+ в периферической крови и ткани щитовидной железы соответствует контрольным значениям, а пониженное относительное число регуляторных T-лимфоцитов в периферической крови соответствует их уровню в ткани щитовидной железы [13]. В то же время при более продолжительном периоде сохранения состояния медикаментозного эутиреоза в крови увеличивается содержание активированных Т-хелперов и цитотоксических Т-лимфоцитов. Подобный результат изменения фенотипического состава Т-клеток при тиреостатической терапии болезни Грейвса можно объяснить тем, что индуцированная тиамазолом клинико-лабораторная ремиссия болезни Грейвса способствует развитию адаптивных изменений в системе иммунитета уже на раннем этапе лечения заболевания, но не сопровождается полной иммунологической ремиссией. Отсутствие взаимосвязей между показателями тиреоидного статуса, уровнем антител к рТТГ и содержанием хелперных и регуляторных Т-лимфоцитов может быть связано с перекрестными интратиреоидными антигенными стимулами, существованием различных вариантов сплайсинга рТТГ, индуцированной тиамазолом смены титра преобладающих блокирующих или стимулирующих антител к рТТГ и соответствующей трансформацией аутоиммунной реакции [2][3].

Таким образом, длительная консервативная терапия тиамазолом способствует повышению уровня регуляторных Т-лимфоцитов у пациентов с болезнью Грейвса, но не влияет на органоспецифические и генерализованные нарушения их супрессорной функции, причем эти нарушения не зависят от продолжительности эутиреоидного состояния, титра антител к рТТГ и, вероятно, могут поддерживать, и усиливать патологический процесс.

Сопоставление с другими публикациями

Результаты нашего исследования согласуются с работами авторов, которым удалось показать, что влияние высоких доз тиреостатиков на ряд иммунологических параметров не отличается от действия низких доз этих лекарственных средств, а основным фактором, от которого зависят иммунологические показатели, является гипертиреоз [14]. Такое влияние тиреостатических препаратов на клетки щитовидной железы, нормализующее ее функцию и восстанавливающее эутиреоз, не объясняет механизм персистенции иммунной реакции, хотя и препятствует стимулирующему действию ТТГ. В нескольких исследованиях сообщалось, что фенотипический состав лимфоцитов периферической крови отражает аутоиммунитет в щитовидной железе как выражение генерализованной активации иммунной системы у пациентов с гипертиреозом аутоиммунного генеза [8][15]. Позднее было показано, что тиреоидные антигены активируют цитотоксические Т-клетки (CD3+CD8+) пациентов с болезнью Грейвса значительно слабее, чем соответствующие клетки здоровых доноров [6]. Кроме того, в более ранних работах сообщалось, что антиген рТТГ гораздо слабее активирует CD3+CD8+-клетки пациентов с болезнью Грейвса, чем CD3+CD8+-лимфоциты здоровых лиц, больных хроническим аутоиммунным тиреоидитом, нетоксическим зобом и сахарным диабетом 1 типа [16].

Сохраняющиеся изменения в фенотипическом составе Т-лимфоцитов, несмотря на поддержание эутиреоидного состояния при лечении тиамазолом более 12 мес, которые были показаны в настоящем исследовании, позволяют утверждать, что высокий уровень CD3+CD4+CD25+ в периферической крови пациентов с болезнью Грейвса может способствовать миграции Th1-клеток из периферической крови в пораженную щитовидную железу. Что, в свою очередь, приводит к усилению презентации тиреоидных антигенов, способствуя дальнейшей активации T-хелперов. При этом субпопуляция CD3+CD4+ может быть источником продукции ИФН-γ, который является негативным регулятором гемопоэза и отражает преобладание Th1-, а не Th2-типа иммунного ответа в начальной фазе болезни Грейвса [12]. Полученные данные существенно дополняют результаты исследования соотношения хемокинов CXCR3/CCR4, указывающего на баланс Th1/Th2, в котором было показано значительное снижение вышеуказанного соотношения после лечения метимазолом по сравнению с исходным уровнем, причем соотношение было значительно ниже на 24-й, чем на 12-й неделе лечения [17]. Авторы делают вывод о том, что при лечении тиреостатическими препаратами происходит постепенный переход от доминирования Th1 к доминированию Th2 иммунного ответа. Ряд современных данных указывает на первичный дефект в снижении количества и функции неспецифических регуляторных Т-лимфоцитов в гипертиреоидной фазе болезни Грейвса, преодоление которого происходит по мере развития медикаментозного эутиреоза и нормализации соотношения Th1/Th2.

Многие авторы подчеркивают, что в основе рецидива гипертиреоза при болезни Грейвса лежит частичное сохранение нарушенной генерализованной и антигенспецифичной супрессорной функции регуляторных Т-лимфоцитов, которая не зависит от гипер-, гипо- или эутиреоидного состояния больных [5][18]. Более раннее сравнительное исследование с использованием иммуногистохимии аспиратов щитовидной железы пациентов с болезнью Грейвса и образцов здоровой ткани показало, что антитиреоидные препараты могут усиливать экспрессию Fas-лиганда (FasL) на мембране тиреоцитов с последующей активацией Fas-индуцированного апоптоза этих клеток [19]. Причем было обнаружено, что обработанные в условиях in vitro метимазолом тиреоциты индуцировали FasL-зависимый апоптоз в культивируемых лимфоцитах, в то время как обработка метимазолом лимфоцитов, выращенных в отсутствие тиреоцитов, не оказывала такого эффекта. Таким образом, FasL высоко экспрессируется в фолликулярных клетках щитовидной железы у пациентов с болезнью Грейвса, получающих тиреостатические препараты, и может способствовать прямому иммуномодулирующему эффекту тионамидов.

Современные представления свидетельствуют о том, что система Fas-FasL является наиболее изученной системой активации гибели клеток в ходе терминации иммунного ответа, а дисрегуляция рецептор-опосредованного апоптоза играет ключевую роль в иммунопатогенезе болезни Грейвса [20]. Настоящее исследование существенно дополняет представления о развитии адаптивного иммунитета при болезни Грейвса на фоне медикаментозного лечения тиамазолом. Было показано, что при приеме тиамазола пациентами с болезнью Грейвса повышение числа Т-клеток, несущих маркер ранней активации (CD25+), наблюдается уже в 1-й фазе терапии и может отражать потенциальный механизм генерализованной иммунологической толерантности тиреоцитов от атаки Т-лимфоцитами. Этот факт приводит к утверждению важной роли Fas-опосредованного апоптоза не только интратиреоидных лимфоцитов, но и периферических аутореактивных Т-лимфоцитов при болезни Грейвса.

Клиническая значимость результатов

Выявленные закономерности в изменении фенотипического состава Т-лимфоцитов периферической крови на фоне тиреостатической терапии свидетельствуют о возможных иммуномодулирующих эффектах длительной терапии тиамазолом на систему адаптивного иммунитета при болезни Грейвса. Полученные результаты позволяют выделить субпопуляцию Т-регуляторных клеток (CD3+CD4+CD127LowCD25High) не только как мишень для этиотропной терапии супрессорного дефекта иммунной системы, но и в качестве возможного маркера иммунологической ремиссии заболевания перед завершением курса длительной тиреостатической терапии в клинической практике.

Ограничения исследования

К возможным ограничениям настоящего исследования популяции пациентов с болезнью Грейвса можно отнести иммуногенетические аспекты уже имеющихся нарушений в иммунной системе, а также генетические аномалии тиреоцитов. Клиническая значимость иммуномодулирующего эффекта тиамазола может быть смещена в зависимости от силы дефекта антигеноспецифической иммуносупрессии в сочетании с неспецифическим влиянием факторов внешней среды в каждом конкретном случае заболевания.

Направления дальнейших исследований

Необходимы дополнительные иммуногенетические исследования для персонализированного стратегического планирования длительной консервативной терапии тиамазолом в зависимости от ассоциации экспрессии HLA-DR и специфического дефекта иммунорегуляции, обусловленного аномалией генов, кодирующих антигенпрезентирующие молекулы. Перспективными представляются исследования по изучению хемилюминесцентной активности и внутриклеточного метаболизма клеток иммунной системы в аспекте ингибирования образования свободных кислородных радикалов при длительной тиреостатической терапии, которые не только дополнят фундаментальные аспекты состояния клеточноопосредованного иммунитета при болезни Грейвса, но и помогут определить терапевтические мишени для этиотропной терапии.

## ЗАКЛЮЧЕНИЕ

Проведено клинико-иммунологическое обследование пациентов с болезнью Грейвса в зависимости от продолжительности стойкого медикаментозного эутиреоза. Охарактеризованы изменения в фенотипическом составе Т-лимфоцитов периферической крови с учетом компенсации гипертиреоза и уровня антител к рТТГ. Установлены механизмы адаптационных изменений в иммунной системе больных болезнью Грейвса при длительной тиреостатической терапии, независимо от титра циркулирующих антител к рТТГ и продолжительности эутиреоидного состояния. В зависимости от продолжительности медикаментозного эутиреоза растет популяция регуляторных Т-лимфоцитов с фенотипом CD3+CD4+CD127LowCD25High как в группе больных со стойким медикаментозным эутиреозом от 5 до 8 мес, так и у пациентов с длительностью эутиреоза от 9 до 12 мес. Повышение количества активированных Т-хелперов с фенотипом CD3+CD4+CD25+ сохраняется у пациентов с длительностью медикаментозного эутиреоза более года. При болезни Грейвса с продолжительностью медикаментозного эутиреоза более 12 мес отмечается компенсаторное повышение регуляторных Т-лимфоцитов, а общее количество Т-хелперов восстанавливается до уровня контроля. Количество Treg в периферической крови у пациентов с болезнью Грейвса повышается с увеличением длительности медикаментозного эутиреоза, но, несмотря на увеличение регуляторных Т-лимфоцитов, процент активированных Th-клеток остается высоким независимо от продолжительности эутиреоидного состояния при консервативном лечении тиамазолом (рис. 1).

**Figure:**
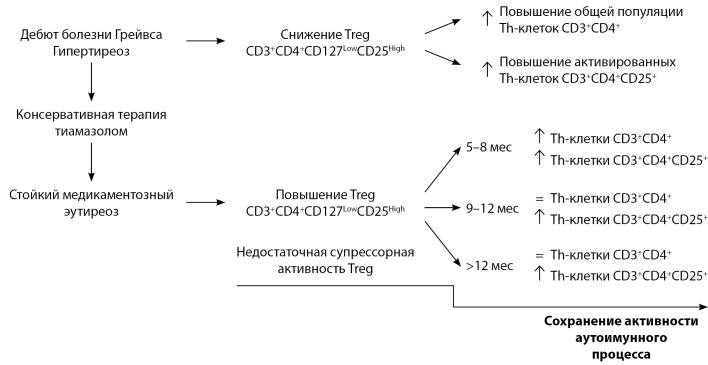
Рис. 1. Изменение соотношения Th и Treg в периферической крови у пациентов с болезнью Грейвса в зависимости от продолжительности медикаментозного эутиреоза при консервативной терапии тиамазолом. Примечание. Cтрелками указаны изменения относительно контрольных значений. “=” — cоответствие контрольному диапазону.

Выявленные изменения в фенотипическом составе Т-лимфоцитов при длительном лечении тиамазолом демонстрируют необходимость разработки таргетной терапии, направленной на восстановление дефекта неспецифических и антигеноспецифических регуляторных Т-лимфоцитов, вследствие которого супрессорная функция регуляторных Т-клеток при болезни Грейвса активируется в меньшей степени.

## ДОПОЛНИТЕЛЬНАЯ ИНФОРМАЦИЯ

Согласие пациентов. Все исследования выполнены с информированного согласия испытуемых и в соответствии с Хельсинкской декларацией Всемирной ассоциации «Этические принципы проведения научных медицинских исследований с участием человека» с поправками 2013 г. и «Правилами клинической практики в Российской Федерации», утвержденными Приказом Минздрава РФ от 19.06.2003 г. № 266.

Источники финансирования. Исследование выполнялось на базе лаборатории молекулярно-клеточной физиологии и патологии Федерального исследовательского центра «Красноярский научный центр Сибирского отделения Российской академии наук», обособленное подразделение «НИИ медицинских проблем Севера».

Конфликт интересов. Авторы декларируют отсутствие явных и потенциальных конфликтов интересов, связанных с содержанием настоящей статьи.

Участие авторов. Все авторы одобрили финальную версию статьи перед публикацией, выразили согласие нести ответственность за все аспекты работы, подразумевающую надлежащее изучение и решение вопросов, связанных с точностью или добросовестностью любой части работы.
